# The Effect of Baduanjin on Promoting the Physical Fitness and Health of Adults

**DOI:** 10.1155/2014/784059

**Published:** 2014-06-23

**Authors:** Ran Li, Li Jin, Ping Hong, Zi-Hong He, Chuan-Ye Huang, Jie-Xiu Zhao, Mei Wang, Ye Tian

**Affiliations:** ^1^China Institute of Sport Science, Beijing 100061, China; ^2^Wuhan Institute of Physical Education, Wuhan, China

## Abstract

The purpose of study was to assess the efficacy of a 16-week Baduanjin qigong training intervention in promoting physical fitness and health for adults. An experimental design was adopted, and subjects were assigned randomly into an experimental group (*n* = 55) and a control group (*n* = 55). In the intervention group comprised of adults, there were no significant variations in blood glucose, blood lipid, blood pressure, heart rate variability, and vital capacity indices. The body weight and body mass index (BMI) dropped in the intervention group. Compared with the control group, the skinfold thicknesses decreased at lower corner of scapula, triceps brachii, and abdomen, with a statistical significance (*P* < 0.001; *P* = 0.005; *P* = 0.003). By comparing the physical fitness indices, it was found that the increase of the results of sit-and-reach test in the intervention group had statistical significance (*P* = 0.001). In conclusion, it was found by our trial that Baduanjin exercise could significantly improve the physical flexibility and subcutaneous adipose accumulation in the healthy adults.

## 1. Introduction

Baduanjin is a qigong which has more than one thousand years of history in China. It is a typical exercise to promote health and the Chinese Health Qigong Association has recommended its generalized application in the community [1]. Baduanjin exercise studied in this paper is compiled by the Chinese Health Qigong Association. This exercise is based on the common rules of Baduanjin exercise, combined with the holistic view and the theory of Qi in traditional Chinese medicine [2, 3]. When practicing Baduanjin exercise, the body maintains a steady gravity center. With the lumbar spine as the axis, the movement of the four limbs is driven. The muscle tension and relaxation are alternating at different parts of the body. In practicing, the mind, body, and breath are required to be smooth and unstrained [3, 4]. Mind in qigong refers to one's mental state and normal consciousness and bodily movements guided by the mind and thoughts. The two form an interactive and interpromotional integrity, characterized by harmony and symmetry shown in all and between every two movements. Baduanjin is noted for its smooth comfortable postures and its movements are performed with profound inner strength. With a concentrated mind followed by a vigorous body, it naturally combines firmness with gentleness, and exercises through the interplay of empty and full [3].

The control of these elements follows a certain sequence. The continuity and mastery of the movement required in practicing Baduanjin exercise cannot be fully achieved unless after a certain period of training. Therefore, the practicing of Baduanjin exercise requires persistence, gradual improvement, and reasonable arrangement of time [3].

As China's traditional form of sport, Baduanjin exercise has gained a wide popularity in China [1, 5]. However, the health promoting value of Baduanjin exercise has not been fully recognized worldwide as that of Taijiquan [6–8]. The positive effects of Baduanjin exercise as a traditional Chinese qigong are yet to be confirmed sufficiently through the randomized controlled trials [4]. Relevant studies at home and abroad are quite limited. Highlighting the movement rhythm and a good control of the movement intensity, Baduanjin can be used as an auxiliary therapy for knee joint arthritis [1, 9]. Due to the requirement for the coordination between the mind and the body in Baduanjin exercise, psychological assessment has been conducted after the intervention [10]. Baduanjin exercise is widely accepted between middle-aged and elderly populations with abnormal blood fats or associated metabolic diseases. For this reason, the effect of Baduanjin exercise in the intervention for abnormal blood fats has been extensively studied [5, 11–13].

Baduanjin is a safe aerobic exercise which features a movement intensity and format in line with the theories of kinetics and physiology. It is different from other types of aerobic exercise in that the practitioners are required to reach the coordination between the mind and the body [1]. A greater emphasis is laid on exercising the body and cultivating the disposition. So far, few studies have been devoted to the health promoting effect of Baduanjin exercise among healthy adults. Most studies concerning the health promoting effect among patients with chronic diseases are limited to the observation of psychological indices and blood lipid indices [5, 10]. But when evaluating a traditional form of qigong in China from the holistic view, the benefits of Baduanjin exercise cannot be fully reflected by a single index. According to the characteristics of Baduanjin exercise, we designed a pilot randomized controlled trial, in which the effects of Baduanjin exercise on the physical fitness and health were analyzed. The tested indices included the indices of psychology, blood lipids, body shape, physical functions, and physical fitness. The trial was designed with the intention of fully reflecting the health promoting effect of Baduanjin exercise on healthy adults, who practiced Baduanjin for 16 weeks.

## 2. Materials and Methods

### 2.1. Participants

From October 2012 to February 2013, the subjects were recruited from the surrounding areas of Wuhan Sports University. The subjects were aged from 20 to 59 years and were local residents. They agreed to take part in the tests designed by the Institute and practice Baduanjin for 16 weeks. The subjects were physically healthy, with no cardiovascular diseases, diabetes, or abnormal glucose tolerance or other acute or chronic diseases that might affect the sports activity. These subjects had no regular physical exercises of moderate intensity. This study was carried out in accordance with The Code of Ethics of the World Medical Association (Declaration of Helsinki) for experiments involving humans. Ethical approval was obtained from the Ethics Committee of the China Institute of Sport Science. The researcher explained the study purpose, procedure, and right of free withdrawal to all participants willing to participate in this study. Informed consent was also obtained.

### 2.2. Trial Design

In this pilot and randomized controlled trial, several evaluation indices characterizing different aspects of physical fitness and health were selected. As no previous study is available, the sample size was estimated based on the main effect indicators and the measurement data were collected in the preliminary experiment of fifteen volunteers before the trial (Table 1). When Type I error was 5% (*α* = 0.05), Type II error was 80% (*β* = 0.20), respectively, to detect a 15% mean difference at end of intervention. The sample size required was calculated according to the formula *n*_1_ = *n*_2_ = 2(*Z*_*α*/2_ + *Z*_*β*_)^2^ *δ* ^2^/*σ* ^2^ (Table 1). Therefore, the intervention group and the control group had 55 subjects, respectively, with the dropout rate controlled at 20%.

Randomization was performed by computer-generated random allocation sequence by simple randomization and undertaken by a research assistant, from China Institute of Sport Science, not involved in recruitment to ensure allocation concealment. The random assignment occurred after completion of baseline questionnaires and assessment. The search assistant who collected and entered study data remained blinded to group allocation throughout the study.

### 2.3. Intervention Group

The subjects in the intervention group undertook the learning of Baduanjin exercise two weeks prior to the intervention. The training was under the guidance of professional coach. The learning status of the subjects was evaluated before the intervention began. The 55 subjects that could practice the whole Baduanjin exercise were enrolled in the intervention program: the subjects practiced Baduanjin exercise 3 times or above each week, 30–60 minutes each time. The subjects were required to complete the program in the fixed site and in a collective manner, with special personnel recording the attendance and providing guidance.

### 2.4. Descriptions of the Baduanjin Routines

The whole set of Baduanjin exercise consists of 9 postures: (1) ready position, (2) holding the hands high with palms up to regulate the internal organs, (3) posing like an archer shooting on left and right sides, (4) holding one arm aloft to regulate the functions of the spleen and stomach, (5) looking backwards to prevent sickness and strain, (6) swinging the head and lowering the body to relieve stress, (7) moving the hands down the back and legs and touching the feet to strengthen the kidneys, (8) thrusting the fists and making the eyes glare to enhance strength, and (9) raising and lowering the heels to cure diseases [3].

### 2.5. Control Group

The subjects in the control group were instructed not to take part in any exercise classes or regular physical exercise during the study period. They were informed to maintain their original lifestyle in the intervention period. Sixteen weeks later, all participants in the control group got the 2-week Baduanjin exercise training under the guidance of professional coach.

### 2.6. Study Measures

The measurements and survey were performed twice for all subjects, as the baseline and end line survey, respectively. Before the subjects entered into the cohort, they were already informed of the contents of measurement and signed the informed consent. The research assistants in Wuhan Institute of Physical Education who collected and entered study data remained blinded to group allocation throughout the study.

Measurement of four blood lipid indices was as follows. The blood samples were collected before meal in the morning. The indices measured included serum total cholesterol (TC), serum triglyceride (TG), serum low-density lipoprotein (LDL), and serum high-density lipoprotein (HDL). The detection kits were provided by Roche; the equipment was Automatic Biochemistry Analyzer (7020, Hitachi, Japan).

Measurement of body shape was as follows: the indices of body shape measured included body height, weight, chest circumference, waist circumference, hip circumference, skinfold thickness of inferior angle of the scapula, skinfold thickness of abdomen, and skinfold thickness of triceps brachii.

Measurement of physical fitness included the following. (1) Sit-and-reach test (SR): the subjects sat on the cushion, with two legs stretching forward; the soles of the feet were pressed together and placed flat against the baffle of the analyzer, with the tiptoes naturally apart. During the test, the subjects had hands closed together, with palm reaching downwards. The knee joints stretched straight, and the body bent forward. The tip of the middle finger pushed the vernier on the analyzer forward. (2) Eye-closed and single-legged standing test (ES): the subjects were required to close the eyes and lift up one foot. When the supporting foot moved or the lifted foot touched the ground, the test was over, and the time of single-legged standing was recorded. (3) Measurement method of aerobic endurance: the increasing exercise load method was used. The subjects were required to maintain a 60 rpm rotational speed on the bicycle ergometer (ergoselect 100, Ergoline, Germany). Both the initial load and increasing rate were 25 W for males and 20 W for females. The load of each level lasted for 2 min. After 2 min, the load of next level was applied without rest. The load was increased continuously at this rate until the subjects were exhausted. The gas analyzer (MetaMax 3B, Cortex Biophysik, Germany) was used in the whole process for acquisition of signals, including maximal oxygen uptake (VO_2_max), maximal heart rate (HRmax), and maximal ventilation (VEmax).

Measurement of physical functions was as follows: the indices measured included blood pressure, heart rate variability (HRV), vital capacity, and time vital capacity. In the vital capacity measurement, the one-time maximum expiratory volume of the subjects was measured. The time vital capacity was measured by portable lung function meter (CSTF-FH, ZhongTiTongFang, China). The parameters measured in spirometry were vital capacity (VC), forced vital capacity (FVC), forced expiratory volume (FEV) at timed intervals of 0.5, 1.0 (FEV1), 2.0, and 3.0 seconds, peak expiratory flow (PEF), and forced expiratory time (PET). The heart rate variability was measured by Polar RS800cx (Polar, Finland) during the 10-minute recording. The following time domain parameters of HRV were mean RR intervals (Mean R-R). Frequency domain variables included total power, very low (VLF: 0–0.04 Hz), low (LF: 0.04–0.15 Hz), and high (HF: 0.15–0.40 Hz) frequencies. The values for LF and HF were presented as normalized units. Normalization was achieved by dividing the spectral power of LF or HF by the total power minus the VLF. The LF/HF ratio was achieved by dividing the LF by the HF in normalized units.

### 2.7. Study Questionnaire

All participants completed a questionnaire about general information about lifestyle and health. The Zung self-rating depression scale (SDS) to measure depression was used at the baseline and end line survey [14, 15]. SDS has 20 items rating the four common characteristics of depression and anxiety. The scores were counted up and multiplied by 1.25 to reach a standardized score.

### 2.8. Data Analysis

Statistical analysis was performed using SPSS 10.0 (SPSS Inc., Chicago, IL, USA). Independent-sample *t*-test was used to test posttest-pretest scores between the experimental and control groups. A two-sided test was used in this study. In all cases, values of *P* < 0.05 were considered statistically significant.

## 3. Results

**Figure 1:**
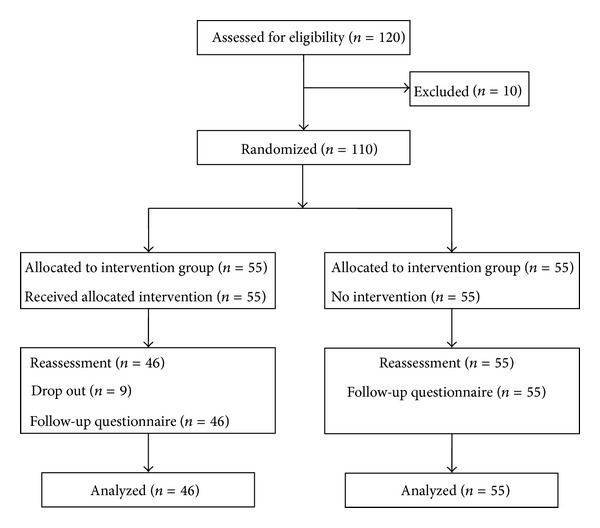
The flow of participants through the trial.

Figure 1 shows the flow of participants through the study. Because ten subjects with suspected chronic diseases were excluded from this trial, the sample consisted of 110 participants (36 men and 74 women) aged 20–59 years, mean age 34.2 years (SD 14.6). The participants in the two groups had similar baseline characteristics (included in Table 2). The 110 subjects were physically healthy, with no acute or chronic disease that might affect the sports activity, and had no regular physical exercises of moderate intensity. Four participants quitted the intervention because they disliked the regular and collective manner. Three participants quitted the intervention without any reasons. Two participants quitted the intervention because of the family vacation. These resulted in completion rates of 100% for the control group and 83.6% for the intervention group in the physical measures and questionnaire, respectively.

### 3.1. Intervention Adherence and Participant Retention, Intervention Enjoyment/Satisfaction, and Adverse Events

Of 55 subjects initially recruited into the intervention group, 46 subjects regularly participated in the 16-week Baduanjin exercise training program and the baseline and end line surveys. The loss rate was 16.4%. In addition to Baduanjin exercise training 3 times each week, 30–60 minutes each time, the subjects in the intervention group did not take part in other regular physical exercises. The number of trainings attended ranged from 30 to 48. Five people (10.9%) attended at least 30 of the trainings. Forty-one people (89.1%) attended all 48 trainings. The reasons given for nonattendance included illness (*n* = 3), going away on holidays (*n* = 1), and lack of time (*n* = 1). No subject presented any discomfort related to the practicing of Baduanjin exercise during the intervention period. The satisfaction of subjects towards Baduanjin exercise in the intervention group was 100%. This suggested that Baduanjin exercise had a high suitability as an exercise intervention among the population.

### 3.2. Effect of Intervention on Outcome Measures

In the intervention group comprised of adults, there were no significant variations in blood glucose, blood lipid, blood pressure, heart rate variability, and vital capacity indices (Tables 3 and 5). Compared with the control group, the body weight and body mass index (BMI) dropped in the intervention group, but the difference was of no statistical significance. However, compared with the control group, the skinfold thicknesses at three typical positions (lower corner of scapula, triceps brachii, and abdomen) decreased, with a statistical significance (Table 4). By comparing the physical fitness indices of the subjects, it was found that the results of sit-and-reach test in the intervention group were increased significantly. However, the control group showed no such changes. The increase of the results of sit-and-reach test in the intervention group had statistical significance (Table 6). There were no other obvious changes in other physical fitness indices. By comparing the results of SDS scores, there was no significant difference in the average scores between the intervention group and control group (Table 7).

## 4. Discussion

The physical flexibility was evaluated by sit-and-reach test. It was found that the practicing of Baduanjin exercise could significantly improve the physical flexibility of the subjects. The improvement of the results of sit-and-reach test reflects the effect of Baduanjin exercise in promoting the motion of shoulder joint and sacroiliac joint. Other studies have also confirmed the improvement of knee joint by Baduanjin exercise [1, 9]. Physical flexibility is closely related to health. The increase of physical flexibility can improve the physical coordination ability, physical power and velocity, and the physical skills. It is crucial for preventing the athletic injuries [16–18]. Therefore, Baduanjin exercise has the benefit of contributing to the physical flexibility, which is also one of the aims in the design of Baduanjin exercise [3]. Compared with other gymnastic exercises, Baduanjin exercise highlights the unification of physical movement and the mind, with great attention paid to the connotation of physical exercise.

In this study, there was no significant difference in the scores of SDS between the intervention group and the control group. One reason for non-significant findings can be explained that the vast majority of participants are healthy. But in 2 subjects who were assessed as having mild depression before the intervention (initial SDS score higher than 50) [19, 20], the initial SDS scores significantly decreased from 61 to 25 and from 54 to 40, respectively. However, the effect of Baduanjin exercise in alleviating depression needs to be further verified. Existing studies have shown that intervention based on Baduanjin exercise could improve the sleeping quality of elderly subjects [10]. It is obvious that the research on the psychological impacts of Baduanjin exercise on the subjects needs to be furthered.

The moderate intensity exercises are beneficial for the improvement of body weight, BMI, and body composition [21–24]. In this study, the decrease in the skinfold thicknesses of the lower corner of scapula, biceps brachii, and abdomen was of statistical significance compared with the control group. However, the changes in body weight, BMI, and waist circumference were of no statistical significance compared with the control group. These results may indicate that although Baduanjin exercise reduces the accumulation of subcutaneous adipose, it is not ideal in controlling the abdominal obesity.

The previous researches have shown that Baduanjin exercise could improve the blood lipids of the subjects [5]. However, our research indicated that Baduanjin exercise did not significantly improve the blood lipids in healthy adults who practiced Baduanjin exercise. It is undeniable that the measurement results are affected by the variations in the subjects, intervention time and period, the choice of seasons for practicing and whether the subjects' lifestyle is controlled [24, 25].

There were no significant variations in blood pressure, heart rate variability, vital capacity, time vital capacity, maximum oxygen uptake, maximum heart rate, and maximum ventilation among the subjects. It is indicated that the practicing of 16-week Baduanjin exercise can hardly improve the heart functions, lung functions, and cardiopulmonary endurance. One reason is that Baduanjin exercise as a safe aerobic exercise may be a moderate intensity exercise [26, 27]. Another reason is that the intervention period in this study was only 16 weeks, which is too short to demonstrate the protective effect of Baduanjin exercise to cardiopulmonary functions, if any [28].

We acknowledge that this study has several limitations. Firstly, the small sample size makes it difficult to draw conclusions about wider implications of the results. However, this was designed to be a pilot trial to assess protective effect of Baduanjin exercise. Secondly, the high rates of adherence may have been biased as the Baduanjin trainings were offered free of charge and cost may be a common barrier to exercise participation. Finally, the intervention was limited to only 16 weeks of Baduanjin, which may not be enough to demonstrate all the protective effects of Baduanjin exercise. Future studies should include a longer duration of intervention.

## 5. Conclusion

It was found by our trial that Baduanjin exercise could significantly improve the physical flexibility and subcutaneous adipose accumulation in the healthy adults. Baduanjin exercise is a form of sports highlighting physical flexibility and mind-body coordination. Given the limitations of the present study, the psychological impacts of Baduanjin exercise should be further confirmed.

## Figures and Tables

**Table 1:** The data of the preliminary experiment.

Characteristic	Mean	SD	Sample size
Weight (kg)	59.3	10.8	22
Waist (cm)	78.5	8.1	7
Inferior angle of the scapula (mm)	21.1	5.1	40
Triceps (mm)	19.7	5.0	44
Abdomen (mm)	26.9	6.1	35
Systolic (mmHg)	108.4	14.3	12
Diastolic (mmHg)	68.4	9.2	13
VC (mL)	3178	579	22
FVC (L)	2.8	0.7	42
Mean R-R (ms)	854.3	243	55
SR (cm)	11.9	3.3	52
VO_2_max (mL/kg*·*min)	28.3	5.5	25

**Table 2:** Baseline characteristics of participants in intervention and control groups.

Characteristic	Intervention	Control	*t* value	*P* value
Male	17	19		0.420^#^
Female	38	36		
Age	35.5 (16.0)	32.9 (13.0)	0.944	0.347∗
BMI	23.2 (3.3)	22.5 (3.1)	1.162	0.248∗
TG (mmol/L)	1.06 (0.58)	1.11 (0.96)	−0.337	0.737∗
TC (mmol/L)	4.54 (0.99)	4.30 (0.82)	1.372	0.173∗
LDL (mmol/L)	2.86 (0.86)	2.62 (0.72)	1.513	0.133∗
HDL (mmol/L)	1.59 (0.40)	1.54 (0.35)	0.688	0.493∗
Weight (kg)	63.5 (11.2)	61.4 (11.8)	0.939	0.350∗
Chest (cm)	89.9 (7.7)	88.8 (8.0)	0.683	0.496∗
Waist (cm)	83.1 (10.1)	79.9 (10.6)	1.580	0.117∗
Hip (cm)	94.8 (7.4)	94.5 (5.7)	0.276	0.783∗
Inferior angle of the scapula (mm)	17.7 (5.0)	16.2 (4.9)	1.632	0.106∗
Triceps (mm)	17.0 (6.2)	15.4 (5.0)	1.511	0.134∗
Abdomen (mm)	19.2 (5.8)	16.4 (5.4)	2.568	0.012∗
Systolic (mmHg)	117.3 (12.3)	114.4 (13.3)	1.079	0.284∗
Diastolic (mmHg)	75.4 (8.8)	71.0 (8.7)	2.335	0.022∗
Mean R-R (ms)	857.1 (114.4)	827.1 (134.4)	1.256	0.212∗
LF%	47.2 (22.6)	45.8 (20.7)	0.326	0.745∗
HF%	172.0 (905.1)	54.6 (20.4)	0.961	0.339∗
LF/HF	1.5 (1.7)	1.4 (2.0)	0.105	0.917∗
VC (L)	3,091.6 (1,011.4)	3,120.4 (791.4)	−0.163	0.871∗
FVC (L)	3.1 (1.0)	3.1 (0.8)	−0.145	0.885∗
FEV1 (L)	3.0 (1.0)	2.8 (0.7)	0.641	0.523∗
FEV1%	90.8 (6.8)	87.5 (15.6)	1.360	0.177∗
PEF (L/s)	5.9 (2.0)	6.1 (2.1)	−0.366	0.715∗
PET (s)	1.5 (0.5)	1.8 (1.1)	−1.819	0.072∗
SR (cm)	15.3 (8.7)	13.1 (11.2)	1.171	0.244∗
ES (s)	39.8 (57.7)	29.4 (24.0)	1.211	0.229∗
VO_2_max (mL/kg*·*min)	28.9 (10.4)	27.7 (7.7)	0.688	0.493∗
HRmax (1/min)	161.1 (32.9)	170.1 (18.7)	−1.744	0.084∗
VEmax (L/min)	60.9 (28.9)	56.7 (15.5)	0.946	0.346∗
SDS	41.5 (11.1)	47.9 (12.0)	−2.900	0.005∗

^#^The chi-square test was used to examine the homogeneity. ∗The *t*-test was used to examine the homogeneity.

**Table 3:** Comparison of blood lipid indices.

Characteristic	Baseline				Follow-up				Posttest-pretest scores				
	Intervention		Control		Intervention		Control		Intervention-control				
	Mean	SD	Mean	SD	Mean	SD	Mean	SD	Mean	95% CI		*t* value	*P* value
TG (mmol/L)	1.05	0.57	1.11	0.96	1.20	0.54	1.26	0.83	−0.07	−0.26	0.12	−0.713	0.478
TC (mmol/L)	4.65	1.01	4.30	0.82	4.23	0.96	4.12	0.97	−0.21	−0.49	0.07	−1.460	0.148
LDL (mmol/L)	2.91	0.89	2.62	0.72	2.39	0.80	2.30	0.74	−0.14	−0.37	0.10	−1.170	0.245
HDL (mmol/L)	1.66	0.40	1.54	0.35	1.54	0.38	1.50	0.35	−0.09	−0.18	0.01	−1.779	0.079

**Table 4:** Comparison of body shape indices.

Characteristic	Baseline				Follow-up				Posttest-pretest scores				
	Intervention		Control		Intervention		Control		Intervention-control				
	Mean	SD	Mean	SD	Mean	SD	Mean	SD	Mean	95% CI		*t* value	*P* value
Weight (kg)	61.1	9.2	61.4	11.8	59.2	9.0	60.3	11.7	−0.5	−1.2	0.2	−1.453	0.150
BMI	22.8	2.9	22.5	3.1	22.0	2.6	22.0	3.2	−0.2	−0.7	0.4	−0.595	0.554
Chest (cm)	88.8	6.8	88.8	8.0	89.0	6.7	88.7	12.4	0.2	−1.1	4.9	1.288	0.201
Waist (cm)	82.1	9.7	79.9	10.6	83.3	9.1	83.8	9.3	0.8	−3.0	2.3	−0.287	0.775
Hip (cm)	94.8	5.7	94.5	5.7	94.2	5.2	95.7	5.3	−1.2	−2.5	0.2	−1.688	0.095
Inferior angle of the scapula (mm)	18.0	4.9	16.2	4.9	14.3	3.8	17.0	6.6	−4.0	−6.0	−1.9	−3.887	0.000
Triceps (mm)	18.1	6.1	15.4	5.1	14.7	4.1	15.3	5.2	−3.4	−5.7	−1.1	−2.894	0.005
Abdomen (mm)	19.5	5.4	16.4	5.4	19.9	5.5	20.3	7.3	−3.3	−5.4	−1.2	−3.092	0.003

**Table 5:** Comparison of body function indices.

Characteristic	Baseline				Follow-up				Posttest-pretest scores				
	Intervention		Control		Intervention		Control		Intervention-control				
	Mean	SD	Mean	SD	Mean	SD	Mean	SD	Mean	95% CI		*t* value	*P* value
Systolic (mmHg)	113.0	7.2	111.0	10.5	110.4	8.8	111.0	10.0	−0.4	−6.3	5.6	−0.119	0.905
Diastolic (mmHg)	73.8	7.4	70.6	8.3	72.3	8.9	74.9	6.9	−4.7	−9.3	−0.1	−2.015	0.048
Mean R-R (ms)	860.2	115.3	827.1	134.4	851.4	121.2	815.9	135.7	2.4	−53.5	58.4	0.086	0.931
LF%	48.6	19.4	45.8	20.7	52.2	18.0	52.7	21.1	−3.2	−13.8	7.5	−0.588	0.558
HF%	51.0	19.5	54.6	20.4	47.9	17.9	47.3	21.1	4.4	−6.2	15.1	0.827	0.411
LF/HF	1.5	1.7	1.4	2.0	1.6	1.5	1.7	1.4	−0.2	−1.1	0.7	−0.434	0.666
VC (L)	2897.4	914.4	3120.4	791.4	2996.9	987.3	3192.7	686.6	53.7	−84.1	191.5	0.774	0.441
FVC (L)	2.9	0.9	3.1	0.8	2.9	0.9	3.1	0.6	0.0	−0.2	0.2	−0.060	0.952
FEV1 (L)	2.8	0.9	2.8	0.7	2.6	0.9	2.7	0.6	−0.1	−0.3	0.1	−0.873	0.385
FEV1%	90.6	7.4	90.1	6.6	87.8	10.6	85.7	10.3	−0.6	−4.7	3.5	−0.280	0.780
PEF (L/s)	5.6	1.8	6.1	2.1	4.9	1.8	5.2	2.3	0.3	−0.3	0.9	0.910	0.365
PET (s)	1.5	0.5	1.8	1.1	1.7	0.6	1.9	0.7	0.1	−0.2	0.5	0.876	0.383

**Table 6:** Comparison of physical fitness indices.

Characteristic	Baseline				Follow-up				Posttest-pretest scores				
	Intervention		Control		Intervention		Control		Intervention-control				
	Mean	SD	Mean	SD	Mean	SD	Mean	SD	Mean	95% CI		*t* value	*P* value
SR (cm)	15.3	8.1	12.7	8.7	17.3	8.6	12.9	8.1	3.5	1.5	5.5	3.464	0.001
ES (s)	31.1	28.7	29.4	24.0	43.7	43.1	43.8	37.3	−21.1	−46.9	4.6	−1.630	0.106
VO_2_max (mL/kg*·*min)	27.8	10.2	27.7	7.7	29.3	11.8	28.1	8.1	1.1	−2.2	4.4	0.658	0.512
HRmax (1/min)	161.8	27.1	170.1	18.7	162.4	24.3	163.8	21.2	6.9	0.2	13.5	2.031	0.045
VEmax (L/min)	52.4	22.4	56.7	15.7	56.8	22.6	56.0	16.3	5.2	−0.2	10.6	1.913	0.059

**Table 7:** Comparison of scores from self-rating depression scale (SDS).

Characteristic	Baseline				Follow-up				Posttest-pretest scores				
	Intervention		Control		Intervention		Control		Intervention-control				
	Mean	SD	Mean	SD	Mean	SD	Mean	SD	Mean	95% CI		*t* value	*P* value
SDS	34.1	9.3	39.9	10	37.1	9.1	40.7	11	2.07	−3.34	7.48	0.759	0.450
